# Dietary Patterns at the Individual Level through a Nutritional and Environmental Approach: The Case Study of a School Canteen

**DOI:** 10.3390/foods11071008

**Published:** 2022-03-30

**Authors:** Cristiana Peano, Vincenzo Girgenti, Savino Sciascia, Ettore Barone, Francesco Sottile

**Affiliations:** 1 Department of Agricultural, Forest and Food Sciences (DISAFA), University of Torino, Largo Paolo Braccini 2, 10095 Grugliasco, Italy; cristiana.peano@unito.it (C.P.); vincenzogirgenti@gmail.com (V.G.); 2 Unesco Chair in Sustainable Development and Territory Management, University of Turin, 10124 Torino, Italy; savino.sciascia@unito.it; 3 Department of Scienze Cliniche e Biologiche, University of Torino, Via Verdi 8, 10124 Torino, Italy; 4 Department of Agricultural, Food and Forest Sciences (SAAF), University of Palermo, Viale delle Scienze, Edificio 4, 90128 Palermo, Italy; ettore.barone@unipa.it; 5 Department of Architecture (DARCH), University of Palermo, Viale delle Scienze, Edificio 14, 90128 Palermo, Italy

**Keywords:** sustainability, school canteen, life cycle assessment (LCA), nonrenewable energy (NRE)

## Abstract

The public catering sector has important responsibilities in seeking a change toward more sustainable choices for many aspects related to the environmental impacts of their services. The environmental impact of production processes can be studied through life cycle assessment (LCA), which allows a greater awareness of choices and has rarely been applied to catering. In this work, we studied the impacts of two dishes (braised meat and cauliflower meatballs) in a school canteen, their impacts were studied using the daily energy requirement (expressed in kcal) as a functional unit. Global warming potential (GWP) and nonrenewable energy (NRE) were calculated starting from the supply of raw materials up to distribution. Electricity and the act of cooking the meatballs accounted for more than 60% of the measured impact in terms of GWP, whereas, less markedly, they dominated in terms of nonrenewable energy used. In the case of braised meat, the total impact was, however, attributable to the life cycle of the meat (between 60% and 76%) and the consumption of electricity (between 19% and 27%), whereas for all other factors, the contribution was never particularly high. Additionally, a discussion on the correct functional unit to be used proposed the environmental impact of different recipes as an additional criterion for nutritionists during the composition of the menu. An integrated system appears important for changing policies and behaviors and the application of LCA can be a tool capable of contributing to the construction of a holistic instrument of sustainability.

## 1. Introduction

The food system is becoming one of the cornerstones of the major global environmental and health challenges that we are currently facing and plays a decisive role in climate change; it has been estimated that up to 30% global anthropogenic greenhouse gas emissions could be attributable to food production and distribution [1,2,3,4]. Within this system, the catering sector, particularly when linked to the public (hospitals, schools, universities, nursing homes, and prisons), has the potential to be a key player in accelerating positive change and influencing both production and consumption. This is because, as a model, it could improve the food supply chains in terms of its efficiency, implement sustainable production activities, promote sustainable consumption, and influence eating habits [5]. For example, in some Italian cities, measures to make school catering services more sustainable with the double objective of decreasing food waste and improving the nutritional patterns of food regimens have already been implemented. These include the purchase of energy-efficient systems, preferring tap water, transport in environmentally friendly vehicles, and a marked decrease in packaging waste. Further indicators of sustainability impacts associated with catering contracts are the use of eco-friendly cleaning substances and preferring bidders who provide more options in terms of organic or fair-trade products [6].

Lang [7] wrote “the environment is nutrition’s invisible infrastructure, everywhere but nowhere”. The environment is essential for food production; however, when nutritional recommendations are given, they are often the neglected. These approaches are more concerned with production and organization than with nutrition, such as replacing beef with a combination of other foods without a significant effect on the nutrient profile of the diet [8].

These observations might be applicable in some niche contexts. Specifically, in school canteens, among others, one of the limiting factors in the construction of the idea of sustainability in diets [9], for students’ parents and for catering companies, is the price, as recalled by Vanclay et al. [10] and Javier Ribal et al. [11]. In Italy, school catering plays an important role as up to 30% of the total meals distributed by institutional catering are delivered in schools [12,13], where about 50% of Italian kids consume their daily lunch (380 million meals per year). This number could increase, as the economic situation created by the COVID-19 pandemic could lead to a situation in which many children would receive the one meal consumed during the day as the only complete and balanced meal from a nutritional point of view at school [14]. Globally, tailored nutritional guidelines have been disseminated to guide the development of school menus, considering the available body of nutritional science to support healthcare providers and policymakers to establish healthy eating schedules for school lunches. For Italy specifically, in 2010 the Italian Ministry of Health released the national guidelines for school catering [15]. These recommendations aimed to provide guidance in the organization and management of the catering services, to regulate contracts with restauranteurs, and to support the identification of balanced meal requirements in different age groups [16]. The requirement for primary school meals defined that lunch should provide the students with 35% of the daily energy requirement. The daily dietary schedule should guarantee around 15% protein, 30% fat, and 55% carbohydrates [17] so that Italian school catering is in line with the definition of a sustainable diet provided by the Food and Agricultural Organization of the United Nations: “those diets with low environmental impacts which contribute to food and nutrition security and to a healthy life for present and future generations. Sustainable diets are protective and respectful of biodiversity and ecosystems, culturally acceptable, accessible, economically fair and affordable, nutritionally adequate, safe and healthy, while optimizing natural and human resources” [18].

The nutritional aspects must also be associated with environmental ones. As mentioned by Heller and Koeleian [19] and Berardy et al. [20], the LCA can be applied when attempting to quantify the environmental impact of the diet, but it depends on an adequate characterization of a nutritional functional unit for comparative analyses [21]. This implementation can be part of a theoretical framework aiming to create a more comprehensive health assessment. Nutrition ecology conceptually encompasses a synergic approach to food, sustainability, and health [22], and a focus on environmental nutrition [23]. LCA can offer a possible theoretical framework [24] to link consumption patterns to production implications and to integrate environmental impact and nutritional health assessment quantitatively.

Although various diets and the supply chain of the single raw materials that comprise them can be assessed through the use of LCA [20,21], this approach has some limitations when applied at the individual level in assessing the environmental effect of the individual portion and meal. As such, when referring to food studies, some functional units often used for LCA become ineffective, such as when mass is used as a unit that does not respond to a number of aspects related to individual food ingredients. Using LCA at the individual level requires the evaluation of the environmental effects of food by computing it as a functional unit (portion) in order to standardize it [25].

The aim of this work was to propose an analysis model that provides public administrations and catering companies that deal with school catering with a tool that can be used by nutritionists in the composition of menus, combining both environmental and fundamental nutritional criteria. Some authors recently proposed methodologies on similar models with the aim of designing diets and menus with minimal impact but at the same time being nutritionally adequate and simultaneously healthy, acceptable, and affordable [26,27]. We had two specific objectives. The first was to identify, through the use of LCA, the environmental effects associated with the processes of production, administration, and disposal of two dishes (braised beef and cauliflower meatballs) using the daily energy requirement (expressed in kcal) as a functional unit. The second was to address the issue of the validity of the functional unit kcal for an adequate integration between the nutritional and environmental aspects of meals. The LCA tool was chosen because it is still only marginally used in the institutional catering system as reported by Carino et al. [28] and Takacs et al. [5] despite being an interesting method for quantifying environmental impacts and being an indispensable tool for making informed decisions. In China, there is an interesting approach focused mainly on evidence of the increasing food waste in a continuously increasing students’ generation [29]. For canteens, especially school canteens, developing a policy that provides adequate information to pupils and their parents is challenging. Similarly, the environmental sustainability of the final meal and the individual constituents of the meal are often neglected by suppliers. Our study provides data-driven evidence to support the implementation of systems to improve sustainability.

Herewith, we presented a case study based on the school canteens of the Municipality of Bagno a Ripoli.

In detail, we first described the settings and characteristics of our case study. Second, we provided data on the impact analysis assessment with an attempt at switching from the traditional LCA analysis that encompasses a mass-based approach to a functional unit (portion) level. Third, we explored the possibilities of identification of the correct functional unit to be used when considering the environmental impact of different recipes as an additional criterion for nutritionists during the composition of the menu.

## 2. Case Study

In this study, we analyzed two dishes served in the school canteens of the Municipality of Bagno a Ripoli that provide the same caloric intake (160 kcal):

Cauliflower meatballs (4 per serving),

Braised meat (1 slice, 80 g).

We identified the environmental impacts related to the two dishes’ production, administration, and disposal processes. In order to achieve this goal, efforts were made to identify appropriate tools that would allow for performance evaluation. The study was conducted through a quantitative analysis using the LCA approach [30,31]. The analysis and data retrieval occurred at the meal preparation site of SIAF S.p.A. (Servizi Integrati Area Fiorentina, Bagno a Ripoli, Florence Province) and in the cooking center of a primary school in Lilliano (Siena Province). The choice of the two dishes, the cauliflower meatballs and the braised beef, was the result of an ongoing discussion with the nutritionist in charge of the municipality. This ongoing discussion with different stakeholders allowed us to involve the several actors participating in the process and to include the food service company and the representatives of the public institution. This participation proved necessary and fundamental in conducting the study, both for the knowledge related to the nutritional principles and the criteria for the composition of school menus in the various age groups, and for the in-depth knowledge of the processes involved in a catering company collective. The functional unit (FU) defines the performance characteristics of the studied system and provides the reference to which the inputs and outputs refer (the ISO 14044—2006 standard). A mass FU could not be considered in this study, as it does not account for the nutritional level of the meal, an essential aspect for dietary LCAs [24]. We, therefore, used a hypocaloric FU, which adjusts all meals to the same energy level: 160 kcal.

**Figure 1:**
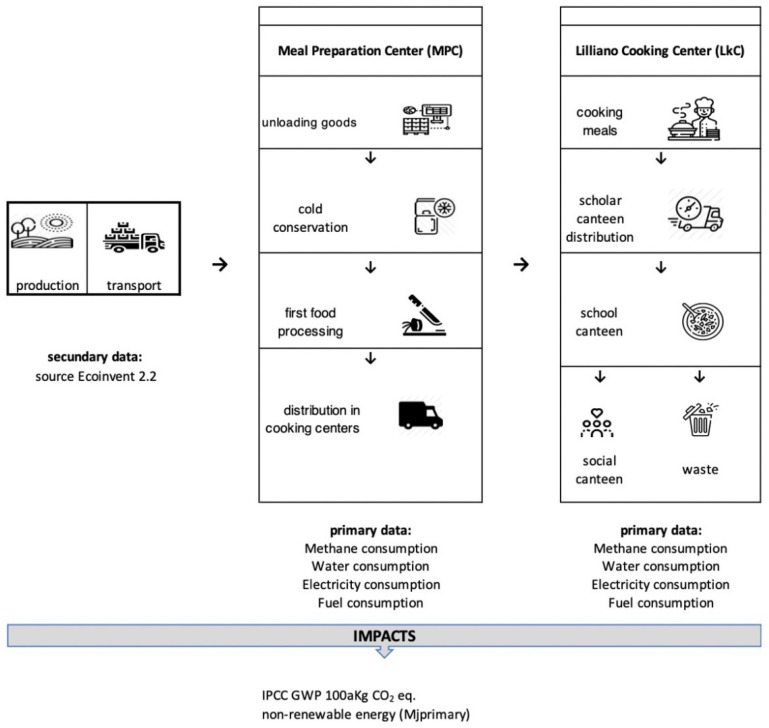
Flow chart of the production phases of the meals for consumption in the school canteen.

The choice of two dishes with the same caloric value allowed us to highlight the environmental impact of the single calorie, linking the nutritional aspects, fundamental in the choices of the composition of school menus, with the environmental effects attributable to the production of the dishes. The FU chosen, set at 160 kcal, followed the complete path reported in Figure 1.

## 3. Material and Methods

The boundaries of the system analyzed were from the cradle to grave [31], all the impacts of the plate were considered in the research, starting from the production phase of the raw materials up to the waste disposal phase.

The food service company, in the preparation of meals related to school catering, had a production phase organized as follows (Figure 1):

- The suppliers deliver the raw materials to the meal preparation center (MPC), located in Via Don Perosi, Bagno a Ripoli, Piemont, Italy.
- At the MPC, the products are stored, and the first preparation of the dish is carried out the day before serving it. All the phases necessary for transformation except the cooking phase, such as washing, cleaning, cutting, and preparation, are then performed.
- The semifinished dishes are transported to the Lilliano Cooking Center (LiCoCe), where the cooking and administration phase is performed at the adjacent school.
- The nonadministered inventories are cut down, packaged, and delivered to Caritas, which delivers them to needy families in the area three times per week.
- The waste is measured and then disposed through separate collection systems for recycling and municipal waste.

The 2% cutoff rule was chosen, making the ingredients and processes associated with an impact higher than this threshold visible in the related graphs. The environmental impact analysis software was SimaPro v. 7.3 PRÈ Consultant 2010. The data related to the raw materials’ production phase were secondary data obtained from the literature and the Eco-Invent database. All of the data related to the internal processes of SIAF and the cooking centers were primary data measured directly and/or taken through semistructured interviews in the year 2019. Specifically, for this assessment, we used input from nurturers and canteen staff to define system boundaries and functional unit quantification.

The method used for the calculation of the results was IMPACT 2002+. The categories chosen to present the environmental impact results were as follows:

- Global warming potential (GWP): Evaluated global warming, obtained by considering among the substances emitted into the air those that contribute to global warming. The mass quantity of each gas was multiplied by a weight factor (different depending on the impact of the single emission and the time frame considered), thus converting all emissions into equivalent kg of CO_2_ as reported by Batlle-Bayer et al. [32].
- Nonrenewable energy (NRE): The primary potential energy contained in the raw materials used was considered. Once used, NRE cannot be restored for further reuse. The unit of measurement of this category was the MJ as reported by Reynolds [33].

The analyzed meals had the compositions reported in Table 1.

Cauliflower Meatballs: The data referring to the waste and the quantity of ingredients and materials used in the preparation and cooking refer to primary data collected in one week in March 2019 during and after the actual administration of the meal in the schools. The data of the quantities of raw materials used are related to the 7000 meatballs prepared, of which 1040 were cooked in the Lilliano Cooking Center (CL) and administered in the school canteen. During cooking, 331 g of baking paper and 1.5 L of oil (with 150 g tin) were used, and in the administration of the 274 meals, paper placemats weighing 4 g were used, for a total of 1096 g. The oven used for cooking the meatballs was a ZANUSSI brand oven model FC-S202G4, and, to calculate the impacts related to cooking, an average cooking time of 20 min was considered, cooking 9 pans of 130 meatballs at a time. After administration, the waste relative only to the dish in question was analyzed, obtaining a weight of 1155 g of waste of the total of 30,960 g of cooked cauliflower meatballs, or 3.7% of the total (See Supplementary Material Tables S1 and S2 for details).

Braised Meat: The data referring to cooking, serving and waste are primary data for the same week of March 2019. Raw material data refer to 1736 servings prepared. Thirteen packages of white wine were used in the cooking process, for a total packaging waste of 585 g. The preparation procedure included an initial cooking in a ZANUSSI oven model FCS202G4, with 60 kg of product at a time and lasting 1 h and 50 min, and a final cooking in a ZANUSSI braising pan model KBR/G3C, lasting about 20 min and with 50 kg of product at a time. During the administration 256 placemats of 4 g each were used, and the analysis of waste after the meal showed a remainder of about 9.5% of the total. Moreover, there were no remnants suitable to be packaged and administered through the Caritas project (See Supplementary Material Tables S1 and S2 for details).

In calculating the impacts of cold storage for both meals, the construction phase of the cold storage facility was not considered (Table 2). The energy associated with each ingredient was then quantified by dividing the total energy consumed in the meal preparation center by the total quantities consumed in the period of September 2018–June 2019. By summing the energy associated with the quantity used for each ingredient, it was possible to calculate the energy consumption related to the 7000 meatballs and 1736 portions of braised meat, and, thus, the energy consumed per serving (Table 3).

As far as transport, the impact of the quantity used in the preparation of the dish was considered. In order to do this, the means of transport used, the load transported, and the kilometers travelled (always within 30 km) were observed for each supplier. In this way it was possible to calculate the impact of 1 kg of product per km travelled and then to report the impact of transport on the quantity of product present in the single portion.

## 4. Results

### 4.1. Impact Assessment

#### 4.1.1. Cauliflower Meatballs

From the characterization chart on the cauliflower meatballs recipe, in the global warming potential (GWP) category, more than 80% of the total impacts were due to three main factors: the use of electricity, cooking the dish, and the life cycle of eggs. Notably, the cooking phase and the consumption of electricity each contributed more than 30% of the total (31% and 33%, respectively), while the eggs had an impact of 23% of the dish’s overall environmental load. Regarding the GWP category, the fourth most significant impact was derived from the consumption of gas (5.8%), followed by the life cycle of the salt (3%). All the other ingredients had a low associated environmental load concerning the environmental impact of the recipe (in kg of CO_2_ eq.), not exceeding 5% of the total. Focusing instead on the impact category of NRE, by observing the graph, we found that the total environmental load was divided more equally between the different raw materials and processes used.

**Figure 2:**
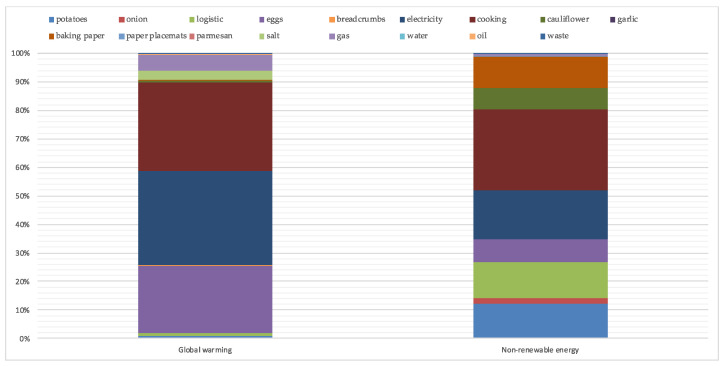
Cauliflower meatballs: impact (percent) considering each ingredient and component.

Even here, three of the greatest impacts were due to the consumption of electricity (18%), the cooking of the product (30%), and the eggs (8.5%), unlike what was observed in the GWP category in this case. The impacts related to the vegetables used in the preparation of the dish (potatoes 13% and cauliflower 8%), the environmental load related to the baking paper used in cooking (12% of the total), that related to the life cycle of the onion (equal to about 2%) and associated with the transport of the product prepared by the MPC to the Lilliano Cooking Center (LiCoCe) were 6% of the overall impact (Figure 2).

As shown in the graph above, electricity and cooking at the LiCoCe played a primary role in forming the recipe’s total environmental impact. In particular, the percentage impact of cooking was similar in both categories, while electricity had a major impact regarding the GWP category.

The life cycle of eggs otherwise contributed much more significantly to the formation of the GWP’s impact, with a difference of about 15 percentage points (23% in GWP and 8% in NRE). Salt and gas consumption visibly contributed to the formation of the total environmental load in the GWP category, while their impact in the NRE category was low. The opposite paths were those of vegetables used as ingredients (potatoes, onion, and cauliflower), transport from the MPC to LiCoCe, and baking paper used in cooking. All the elements indicated had an almost zero impact in the GWP category, while they significantly affected the overall impact of the recipe in MJ. The other ingredients and processes used, such as breadcrumbs, garlic, parmesan, placemats, water consumption, oil, and waste, had a low impact compared to the total in both categories due to the particular characteristics related to the life cycle of the product, the low quantities used, or associated with a portion.

#### 4.1.2. Braised Meat

**Figure 3:**
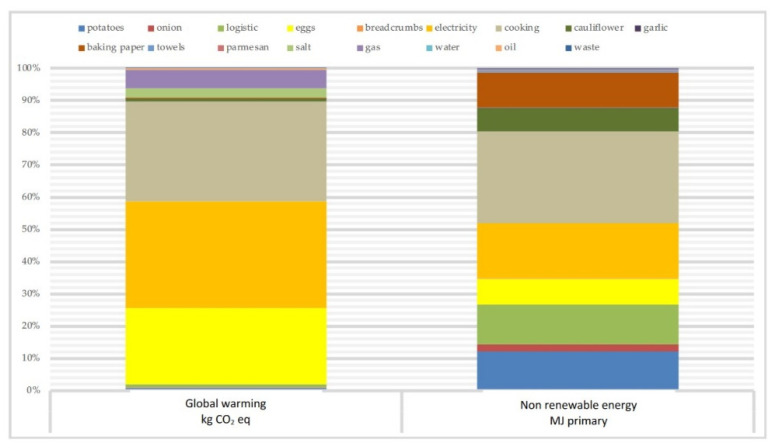
Braised meat: impact (percent) considering the global warming potential and nonrenewable energy of each ingredient and component.

In the second recipe, both categories (GWP and NRE) had a total impact mainly attributable to two factors: the life cycle of the meat and the consumption of electricity. Notably, in the GWP category, meat had an impact of 76% of the total, which fell to 60% in the NRE category. Electricity, consequently, had an associated environmental load of 27% of the total in the NRE category, which dropped to 19% in the GWP. From the graph of the emissions of kg of CO_2_ eq., we observed that, except for cooking at the LiCoCe (which had an impact of 2% of the total), all the other raw materials and all the other processes associated with the recipe did not individually exceed 1% of the impact total (Figure 3).

Therefore, meat was the raw material associated with the highest environmental impact in both impact categories, alone representing 76% of the total environmental load in the GWP category. Except for electricity consumption (which had an impact of more than 15% in both categories), all the other ingredients and processes associated with the preparation, cooking, and administration of braised meat had a low environmental load compared to the total environmental load, which became almost zero in the GWP category, whereas the value was slightly higher in the NRE category (probably due to the difference in the impact of meat in the two categories).

**Figure 4:**
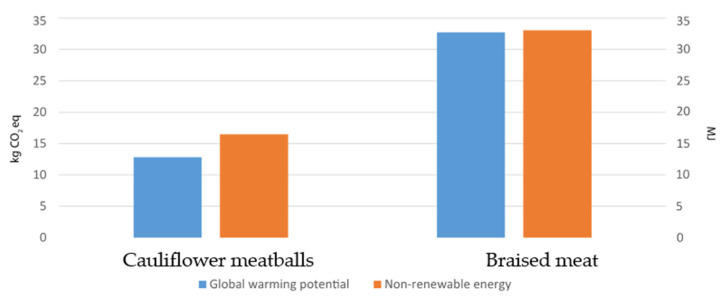
Total impact (global warming potential and nonrenewable energy) of the two meals.

From the data collected, which was limited to the specific case study, we found that the braised dish was associated with a higher environmental load than cauliflower meatballs in both impact categories (32.74 kg of CO_2_ eq. and 33.08 MJ compared to 12.83 kg of CO_2_ eq. and 16.47 MJ, respectively). Since the two dishes provide the same amount of energy (160 kcal), it was possible to calculate the single category’s environmental impact in the two different scenarios, which was equal to 0.204 kg of CO_2_ eq. and 0.206 MJ for braised meat and 0.08 kg of CO_2_ eq. and 0.10 MJ for cauliflower meatballs (Figure 4).

Life cycle assessment (LCA) has been used to measure environmental impacts of consumer products, especially by quantifying the associated resources as well as emissions and other externalities [34]. The CO_2_ emissions reported in this study were found within the value range of previously published ones [35], and matched with the general understanding that a more plant-based diet reduces GHG emissions [24]. In line with this observation, Batlle-Bayer et al. [36] showed that, in addition to being nutritious, a diet based on the Spanish Dietary Guidelines can reduce the greenhouse gas emissions of current dietary patterns through its promotion of plant-based products and reduction in meat consumption. The results of our LCA confirmed, as reported in the reviewed literature on school canteens [37,38], that the food production phase makes the largest contribution in almost all of the categories examined (over 70%) and, in particular, meat products.

The food storage and cooking phases are also important. The energy used for both cooking and storing food (heat and electricity) accounts for a major share of the overall impact on global energy needs and global warming. Environmental improvements in these life cycle stages can be achieved more easily than during the food production stage, such as by consuming electricity, through renewable energy technologies and the purchase of energy-efficient appliances. Therefore, coupling electricity with renewable energy sources could be a key strategy to improve the environmental sustainability of the food sector and, in a broad perspective, to achieve the European Union’s energy and climate goals. In our study, food transportation did not have a high level of impact as we considered a local supply.

Such measures characterize the biosphere and technosphere flows of the life cycle, from the mining and manufacturing to the consumption and disposal of one or more products in various sectors, including [39]. The LCA was originally applied to examine industrial production systems. However, for more than two decades, scholars have been using LCA principles to study agricultural production systems and the associated environmental impacts [40]. Accordingly, for the LCA metrics used in the food systems, the analysis includes land use, water, and energy, and most often considers the global warming potential (GWP) as the dominating environmental impact [24,41].

## 5. Discussion

When comparing products based on environmental impact, LCA has traditionally required a functional unit of a product to be used as a foundation [42]. However, for food LCAs, the functional units are difficult to define and measure. This is due to the variation in the perceived mandatory properties. For example, a functional unit of a food product is based on mass, such as tons or kilograms. Furthermore, using weight as a functional unit to compare two different foods is insufficient because it does not consider how much a person consumes or why someone chooses one product over another. If we compared beef to broccoli by kilogram, this would give an inaccurate measurement because both are usually eaten in various quantities and for various reasons.

Alternative approaches have been suggested for some specific foods. For example, for foods with high protein content a functional unit based on proteins was proposed [20]. While this is obvious, it may not represent the actual bioavailability of amino acids. Although LCA can be a useful tool for comparing foods in terms of environmental impact, it can also be limited by missing real-world issues, such as portion sizes which can vary widely. This issue could possibly be solved using reference amounts customarily consumed (RACCs). In accordance with the Nutrition Labeling and Education Act and the Federal Food, Drug, and Cosmetic Act, the Food and Drug Administration’s (FDA) final rule for establishing the RACC offers a reference to identify actual amounts of typical consumption balanced with recommended portions [20]. The same authors proposed the use of a different indicator (GWPRO) which includes the potential weight of the protein content of the portion and servings size in grams based on RACCs. As expected from a comparison of three different functional units (Table 1), it clearly emerges that on foods such as meat the GWP obtained as a function of the FU varies considerably if only mass is considered or if protein content and RACCs (GWPRO) or kcal are considered, with a GWP similar to each other. With vegetable products such as cauliflower with low protein content (as quantity and quality), the three GWP values do not change significantly.

The GWPRO method is a potential way to include consideration of protein quality and portion size along with environmental performance in the LCA but has limitations depending on the type and protein content of the food and portion size. In any case the FDA-defined RACCs provide manufacturers with the recommended portion sizes for nutrition labeling on foods, which is continuously updated to provide the most accurate and current information [43]. Applying RACCs in connection with LCA provides a better understanding of the environmental impact related to actual food consumption and reflects nutritional aspects (nutritional LCA (nLCA)). A comparison using this methodology is reported in Table 2 where the GWP is based on different parameters of FU calculation.

A limited number of LCA analyses have developed methods that express results that reflect consumer food choices and consumption patterns. While LCA has traditionally applied functional units based on mass or nutrients, impacts have also been expressed in terms of equivalent flows estimated in the biosphere, such as kilograms of carbon dioxide, as well as an aggregate of characteristics in a single score, such as Eco-indicator 99 [44,45]. Additionally, some studies have also attempted to integrate multiple sustainability dimensions into an assessment. In this case, the LCA is combined with a cost–benefit analysis and social impacts are measured by quality-adjusted life years (QALYs) [33]. However, no research has yet investigated an algorithm that sufficiently measures the environmental impact of food while employing nutritional aspects from an individual perspective.

In this setting, we chose the Eco-indicator 99 as a pragmatic tool able to assist designers of a study when reducing the reliance on LCA experts to assess the eco-impacts, as previously discussed by Goedkoop, Effting, and Collignon [46]. Its main utility relies on the ability to provide a set of eco-indicators that can convert the properties of the process into a single eco-impact score. This eco-impact score can be easily used to compare the eco-performance of various design ideas during the concept selection phase [47]. Hence, designers only need to take responsibility for the design itself with less concern for the domain of environmental assessments (e.g., mapping from midpoint to endpoint indicators) [48].

In particular, in this very setting, we found the use of the Eco-indicator 99 convenient because it was specially developed as an end-point indicator, where normalization and weighting have been preset to “simplify the interpretation” of the LCA results [49]. Conversely, other LCA methods (e.g., ReCiPe) vary mainly in terms of the flexibility with which they would have allowed us to enter values for normalization and/or weighting, which in turn might have required further LCA-related analysis

The novelty of our approach relied on the attempt of moving away from the traditional LCA analysis that encompasses a mass-based approach to a functional unit (portion) level. With this aim, we proposed a theoretical framework methodology for incorporating the quality and quantity of macronutrients using RACCs for comparisons based on LCA results, moving from a mass-based unit of analysis to an individual portion level. On the one hand, applying a consistent mass-based approach (e.g., 1 kg of a product) might facilitate the comparisons of the environmental impacts of products across several production systems. However, on the other, the often-applied default of the seemingly neutral weight-based unit might not reflect the nutritional aspects at a functional unit level (portion). In considering food quality (defined as nutrient contents and composition at a portion level), it is imperative to improve our understanding of the complex food–environment connection. All in all, this reflection begins with the realization that mass is not an appropriate functional unit when referring to food. Therefore, we suggest that assessments should be further aligned with the RACCs to strengthen estimates of environmental impacts. This includes a deeper understanding of how much is actually consumed and which macronutrients are actually provided in real-world food portions. The portion sizes provided by the RACCs can be used to calculate a macronutrient score with associated environmental impacts. This will help to align portion sizes with what is actually consumed, providing the analysis with more holistic information that can provide valuable support for decision-making about a healthy and sustainable diet. Taking the above together, the choice of the functional unit is crucial.

Evaluating the environmental impacts of food systems combined with nutritional aspects represents a challenge as food encompasses many functions, with body sustainment being the most apparent. Indeed, quantifying the contribution of each single food component remains an open problem [50]. The FU’s choice reflects the aim and the purpose of the investigation and is influenced by the considered perspective (or consumption or production) with different levels of complexity ranging from the evaluation of agricultural production methods to the comparison of nutrition patterns. Much as [51,52], in a comparative analysis that investigated different food products using a variety of FUs, noticed that the estimated environmental effects changed according to the applied FUs, as was also determined by other authors [53]. One should also remember that food fulfills a variety of functions beyond nutrition, impacting different dimensions such as cultural value, pleasure, taste, and aesthetics. Last but not least, food has a strong impact on individual health. Capturing all these dimensions in a single FU is very unlikely. It is crucial that in each study, the chosen FU and related aspect should be clearly stated, along with the consequences of this selection. On the one hand, the intrinsic quality of a food product plays an important role from the consumer’s perspective. On the other, several additional aspects are similarly noteworthy, such as its origin (as consumers are more likely to prefer domestic or locally produced food), and methods of production. From a theoretical point of view, these aspects should be incorporated in a life cycle sustainability assessment.

A further critical step is planning how to disseminate the information clearly and efficiently about the environmental impact of the consumed food, especially when referring to a target population (e.g., children and their parents).

Hoefkens et al. [54] reported that the format of the nutritional report in food labeling, which can be used by the client, is very relevant [55]. It is important, not only to educate the consumer to prefer the most environmentally sustainable option, but also, from the customer’s point of view, to support canteens and their suppliers to deliver such meals, as shown by Spaargaren. et al. [56] when investigating the scenario of a university canteen.

Ideally, all the previously mentioned aspects, ranging from supplier-specific sustainability to consumer health benefits, should be incorporated in a single quantitative approach. However, for practical reasons, these features had to be dealt with separately. A theoretically unique sustainability score system exclusively developed to meal constituents and their respective supply chains is not feasible on a large scale yet. A requirement that would first need to be fulfilled is the availability of inventory data, i.e., the transparency of the life cycle. Data on the life cycle of food are becoming increasingly available, although this aspect still represents an obstacle when attempting to scale up the process [57]. Policy change is required to create an integrated, regulated, and easy-to-use system to collect and transfer LCAs to envisage the wider applicability of this approach. LCAs should be integrated, as in our experience, with other strategies for political support [58], with many options that have a high impact on the outcome, requiring further adjustments to obtain a holistic sustainability tool [56,59]. It is worth mentioning that consequential impacts are crucial to achieving large-scale dietary changes [60,61]. Other aspects of sustainability, such as the pleasure of eating and costs, should not be overlooked [62]. Menu labeling is instrumental in connecting operators and consumers, further strengthening a relationship towards mutual trust [63]. In addition to communicating with customers, communication with canteen suppliers appears important to provide them with information related to their production’s sustainability and to guide them in the interpretation of the scoring systems. These aspects should be discussed as determinants when a company is being selected for canteen service. It is also believed that transparent communication with suppliers facilitates the sharing of necessary information or data.

## 6. Conclusions

In our framework of the definition of LCA, there were limitations due to arbitrary selections and decisions related to the development process, which are an intrinsic part in any sustainability investigation. However, while we attempted to limit such decisions as much as possible, if unavoidable, they were fully acknowledged and commented upon, even if some inevitable subjectivity in some choices persisted. In our opinion, different changes should have to be implemented to achieve a pragmatic framework that might be easy to use and applicable to a variety of meals, and not prohibitively expensive and time-consuming for the operators involved. The future step is to investigate the feasibility of this framework in a canteen and to assess the public’s opinion.

We have been working in a school canteen, so our approach has necessarily considered the needs for children specifically based on kcal. The modification of the FU, from a quantity to a quality approach (expressed as the calorie intake of the portion), did not change the main result, as already reported in several sector studies, the environmental impact associated with dishes with animal products is greater than the environmental impact of dishes composed mainly of vegetable products [64].

The use of the LCA approach in the collective catering sector and the use, within the reported methodology, of functional units based on the fundamental criteria used in the specific supply chain allowed us to analyze more correctly and consistently the different processes and the different dishes, making it possible for the nutritionists involved in the composition of the menus to consider the environmental impact connected to the different recipes as an additional criterion. An implementation of the entire system’s environmental performance could be achieved by life cycle approaches able to recognize hotspots at different phases of the supply chain [65]. However, the restaurant sector could be supported by combined LCA thinking strategies, as they would be able to assess additional dimensions of sustainability such as nutritional, social, and economic features. Such integrated approaches are currently still lacking. Consequently, upcoming research should aim to combine, in an integrated fashion, different life cycle thinking approaches. Indeed, the implementation of the overall health and nutrition assessment of meals in the context of LCA has the potential of being very relevant in the meal planning process, as it would allow the inclusion of environmental and health dimensions of the different menu options. Sourcing options and the performance of different production approaches will need further investigation, especially when evaluating potential environmental and health effects of organic, integrated, and conventional production strategies. The integration of economic analysis could be informative, as it could provide further insight on the school meal’s price. While promising, our observations need to be confirmed in future studies, especially when considering additional comparative analyses taking into account similar system boundaries and their assumptions.

## Figures and Tables

**Table 1:** Composition of the two different meals analyzed in the case of study.

Braised Meat	g	kcal per 100 g	kcal per Portion	Cauliflower Meatballs	g per Meatball	g per Portion	kcal per 100 g	kcal per Portion
Silverside	100.00	108.00	118.00	Potatoes	11.3	45.2	90	40.680
Olive oil	4.3	900.00	38.70	Cauliflower	11.3	45.2	25	11.33
Salt	1.00	0.00	0.00	Onion	1.4	5.6	26	1.456
White wine	0.12	0.00	0.00	Garlic	0.033	0.133	41	0.054
Celery	2.50	20.00	0.50	Rosemary	0	0.001	96	0.001
Carrot	2.50	35.00	0.875	Egg	0.9	3.6	128	4.608
Onion	5.00	26.00	1.30	Parmesan	0.9	3.6	380	13.680
Water	500.00			Bread crumble	1.150	4.6	351	14.976
				Olive Oil	2.037	8.148	900	73.33
				Salt	0.1	0.4	0	0
Total			160.18	Total				160.12

**Table 2:** Global warming potential (GWP) focused on different FU based on (i) mass (100 g), (ii) GWPRO (Global warming potential ratio) from the literature, (iii) 160 kcal (of beef and cauliflower).

	FU 100 g	FU GWPRO *	FU 160 kcal
kg CO_2_-eq beef	2.5–3	0.16–0.18	0.20
kg CO_2_-eq cauliflower	0.01–0.02	0.02–0.03	0.08

* methodology from Berardy et al. [20].

**Table 3:** Impact per dish and per portion as related to electricity, water, and methane use.

	Cauliflower Meatballs		Braised Meat	
	Total	Portion	Total	Portion
Electricity (Kw)	31.4396	0.018	40.5627	0.0233
Water (m^3^)	1.1337	6.5 × 10^−4^	1.4548	8.4 × 10^−4^
Methane (standard m^3^)	2.4580	1.4 × 10^−3^	3.1839	1.83 × 10^−3^

## Data Availability

Not applicable.

## Supplementary Materials

The following supporting information can be downloaded at https://www.mdpi.com/article/10.3390/foods11071008/s1, Table S1: Ingredients and data on cauliflower meatballs, Table S2: Ingredients and data on braised meat.
