# Supplementary material for: Dietary Patterns at the Individual Level through a Nutritional and Environmental Approach: The Case Study of a School Canteen
Source: Foods. 2022 Mar 30;11(7):1008. doi: 10.3390/foods11071008 (PMC8997873; doi:10.3390/foods11071008)
Supplement: Supplementary file 1 [file foods-11-01008-s001.zip › foods-1663949-supplementary.pdf]

Table S1: ingredients and data on cauliflower meatballs

Ingredients	Supplier	Km from SIAF*	Vehicle	unit	packaging	Waste	Used quantity	cold storage unit	Storage time (day)	Electricity (Kw)	Water (m^3)	Methane (standard m^3)
Garlic	LA TALEA	6.8	<3.5 t	-	-	N/A	700 g	-	-	12.04	0.42	0.67
Cauliflower	TOTI LEONARDO	3	<3.5 t	-	-	22.4%	80 kg	1	1	12.04	0.42	0.67
Onion	NATURITALIA	131	3.5 t	5 kg X2	160 g	-	10 kg	1	2	1.51	0.0055	0.0083
E.V. Olive Oil	FATTORIA RAMERINO	3.5	<3.5 t	5 l x2	300 g	-	9 l	-	-	9.6 x E^{-4}	3.6 x E^{-6}	5.8 x E^{-4}
Bread Crumbs	LA BOTTEGA DEL CASTINO	43	<3.5 t	2 kg x 2	8 g	-	8 kg	-	-	1.21	0.041	0.067
Parmigian	PARMAREGGIO	145	3.5 t	1 kg x 6	60 g	-	6 kg	3	3	0.90	0.031	0.05
Potatoes	NATURITALIA	131	3.5 t	2.5 kg x32	2560 g	-	80 kg	1	2	1.48	0.051	0.081
Rosmary	CHELAZZI MARCO	3	<3.5 t	-	-	N/A	7 g	-	-	0.10	3.7 x E^{-3}	5.4 x E^{-3}
Salt	DAC COMODA	1175	7.5 t	1 kg	10 g	-	1 kg	-	-	0.14	4.6 x E^{-3}	0.011
Eggs	IL POLLO DEL PRATO	35 km	<3.5 t	250 g x 30	1040 g	16.8%	6.25 kg	2	2	0.94	0.033	0.052

Cold storage unit legend

1. Cold storage unit ORTOFRUTTA ZANOTTI- 400 V/3 N/ 50 HZ 2.20 KW- year 1999 maximum storage 2004.4 kg
2. Cold storage unit POULTRY EGGS ZANOTTI-230V/1N/ 50 HZ 0.70 KW- year 1999 maximum storage 973.4 kg
3. Cold storage unit DAIRY PRODUCTS AND FRESH PASTA -230V/1N/ 50 HZ 1.10 KW- year 1999 maximum storage 1555.4 kg

*distance between suppliers and SIAF have been estimated by Google Maps. http://maps.google.it

Table S2: ingredients and data on braised meat

Ingredients	Supplier	Km from SIAF*	Vehicle	unit	packaging	Waste	Used quantity	cold storage unit	Storage time (day)	Electricity (Kw)	Water (m^3)	Methane (standard m^3)
Carrots	CHELAZZI MARCO	3	<3.5 t	4 kg x 2	2	39.5%	4.8 kg	1	4	0.71	0.024	0.039
Onion	NATURITALIA	131	3.5 t	5 kg X2	160 g	-	10 kg	1	2	1.43	0.049	0.0079
E.V. Olive Oil	FATTORIA RAMERINO	3.5	<3.5 t	5 l x2	300 g	-	9 l	-	-	1.40	0.48	0.077
Salt	DAC COMODA	1175	7.5 t	1 kg	20 g	-	1.9 kg	-	-	0.27	9.3 x E^{-3}	0.015
Celery	VIVO BIO	28.6	3.5 t	2 kg x 3	300 g	20%	4.8 kg	1	4	0.71	0.024	0.039
Veal Sirloin	IL FANTINO	40	<3.5 t	-	11.75 g	11%	221 kg	2	5	31.17	1.090	1.735
White Wine	CAVIRO	173	3.5 t	1 l x 23	1.35 kg	-	23 l	-	-	3.39	0.118	0.118

Cold storage unit legend

1. Cold storage unit ORTOFRUTTA ZANOTTI- 400 V/3 N/ 50 HZ 2.20 KW- year 1999 maximum storage 2004.4 kg
2. Cold storage unit RED MEAT-230V/1N/ 50 HZ 1.10 KW- year 1999 maximum storage 1138.4 kg
